# CRISPR/Cas9-mediated editing of *PHYTOENE DESATURASE* gene in onion (*Allium cepa* L.)

**DOI:** 10.3389/fpls.2023.1226911

**Published:** 2023-08-28

**Authors:** Pawan Mainkar, Tushar Kashinath Manape, Viswanathan Satheesh, Sivalingam Anandhan

**Affiliations:** ^1^ ICAR-Directorate of Onion and Garlic Research, Pune, Maharashtra, India; ^2^ Genome Informatics Facility, Office of Biotechnology, Iowa State University, Ames, IA, United States

**Keywords:** *PDS*, onion transformation, CRISPR/Cas9, genome editing, chlorophyll

## Abstract

**Introduction:**

Clustered regularly interspaced short palindromic repeats (CRISPR)/ CRISPR-associated protein 9 (Cas9) is a precise genome editing tool used to introduce genetic modifications in a wide range of crop species. Thus far, there is no report of CRISPR/Cas9-mediated genome editing in onions (*Allium cepa L.*).

**Methods:**

In the present study, we targeted two exons of the gene coding for Phytoene desaturase (*AcPDS*) in onion cv. Bhima Super. The sgRNA-carrying constructs were co-cultivated with 8-week-old embryogenic calli using an *Agrobacterium*-mediated transformation protocol and incubated on the media without hygromycin B selection.

**Results and discussion:**

Out of the total 617 co-cultivated calli, 21 (3.4%) regenerated shoots exhibited three distinct phenotypes: albino, chimeric, and pale green; in comparison to the wild-type non-transformed regenerated shoots. Total chlorophyll content was drastically reduced in albino shoots and significantly decreased in chimeric shoots. Out of the six *Cas9* gene PCR-confirmed regenerated shoots, two exhibited the albino phenotype due to insertions/deletions (InDels) and substitution-based mutations in and around the *AcPDS* target sites. Deep amplicon sequencing revealed a significantly variable InDel frequency between two sgRNAs, ranging from 1.2% to 63.4%, along with a 53.4% substitution frequency. The mutation of the *AcPDS* gene generated a visually detectable albino phenotype, thus confirming the successful editing of the *AcPDS* gene. This is the first time a CRISPR/Cas9-mediated genome editing protocol has been successfully established in onion, with the *AcPDS* gene serving as an example. This study will provide the necessary momentum for researchers to further basic and applied research on onions.

## Introduction

Onion (*Allium cepa* L.) is one of the important horticultural crops cultivated throughout the world. It is known for its distinct flavor and pungency, which are attributed to a variety of sulfur compounds. Onions are beneficial in terms of offering health benefits due to the presence of flavonoids, anthocyanins, fructooligosaccharides, and organosulfur compounds (Goldman, 2011; Bisen and Emerald, 2016; Putnik et al., 2019). Since domestication, productivity, quality, and resilience to biotic and abiotic stresses have been improved in onions using several conventional breeding practices. However, conventional approaches have not produced substantial progress in the genetic improvement of onions and have been hampered by the biennial life cycle, cross-pollinated nature, and severe inbreeding depression (McCallum, 2007; Sudha et al., 2019; Lee et al., 2021). Despite the fact that onions have a large genetic variability, crop improvement has lagged behind other monocot crops (McCallum, 2007; Varshney et al., 2012). The burden of the current global challenges involving food security, increasing human population and changes in climate experienced worldwide lies in the need to improve horticultural crops with high yield and enhanced adaptation to the environment, for which conventional breeding is unlikely to meet the demand (Brewster, 2008; Van den Broeck and Maertens, 2016). To overcome these obstacles, advanced and innovative molecular approaches should be strategically employed to strengthen onion genetic improvement programs.

Several genome editing tools, such as transcription activator-like effector nucleases (TALENs), zinc-finger nucleases (ZFNs), and meganucleases (MNs), have facilitated targeted manipulation of a desired gene in crop plants. However, these methods are expensive, labor-intensive, and require intricate procedures to achieve successful editing (Razzaq et al., 2019). In contrast, CRISPR/Cas9 has emerged as a promising and versatile approach for precise and efficient plant genome editing. It offers an appealing advantage due to its user-friendly design, cost-effectiveness, and adaptability, making it an attractive tool for researchers in the field (Razzaq et al., 2019). The advent of CRISPR/Cas9-targeted genome editing has paved the way toward incorporating beneficial genetic changes for addressing limitations affecting production and utilization. CRISPR/Cas9 genome editing, as a popular genome engineering tool useful for crop improvement, has drawn the attention of breeders and stands out as an essential tool to generate plants with the ability to cope with challenges. The CRISPR/Cas9 system has been applied to a large number of plant species. Still, there are several horticultural crops where the applicability of this technology has not been demonstrated (Koltun et al., 2018), and onion is one among them. A reproducible system for the design, construction, and application of Cas9/guide RNA (gRNA) must be established and evaluated in onions in order to assess the potential of the CRISPR/Cas9 system. The strategy to knock out the *Phytoene desaturase* (*PDS*) gene has been widely used to validate the practicability of the CRISPR/Cas9 system in several plants, including cassava (*Manihot esculenta*) (Odipio et al., 2017), banana cv. Rasthali (*Musa acuminata* cv. Rasthali) (Kaur et al., 2018), and Cavendish banana (*Musa acuminata* subgroup Cavendish) (Naim et al., 2018). Only recently has the onion genome sequence become available (Finkers et al., 2021), thereby enabling the exploration of the potential for CRISPR/Cas9-based genome editing in applied research for this crop.

In the present study, for the first time, we report the successful establishment of a CRISPR/Cas9-based genome editing system by targeting the gene coding for *PDS* of the Indian short-day onion. The insights presented here lay the groundwork for future investigations into gene function and trait development in this important vegetable crop, leveraging the capabilities of the CRISPR/Cas9-based gene editing system.

## Materials and methods

### Isolation and cloning of the *AcPDS* genomic DNA sequence

The annotated *PDS* sequence of *Asparagus officinalis* (Accn. No. XP_020273319.1) was retrieved from the National Centre for Biotechnology Information (NCBI) database and used as a query for the Basic Local Alignment Search Tool-Nucleotide (BLAST*n*) search against the draft genome sequence of onion (https://www.oniongenome.wur.nl/; Finkers et al., 2021) to retrieve *PDS* gene sequence of onion. Primers were designed in the coding region (**Supplementary Table S1**) of retrieved *PDS* sequences of double haploid onion line DHCU066619 *PDS* (accession: GBRN01009640.1) for the isolation of the full-length *PDS* gene from the Indian short-day onion cv. Bhima Super (B. Super). Genomic DNA was isolated from onion cv. B. Super leaf tissue following GenElute Plant Genomic DNA Miniprep Kit protocol (Sigma, USA). PCR amplification was performed using 100 ng of genomic DNA as a template and a gene-specific primer set (*AcPDS* F/*AcPDS* R) with the help of the PrimeSTAR^®^ GXL DNA Polymerase (Takara, Japan), as per the PCR condition described in **Supplementary Table S1**. The amplified PCR product was cloned into a pJET1.2 vector (ThermoScientific, USA) as per the manufacturer’s instructions. Clones were sequenced by Sanger’s platform at Eurofins Genomics India Pvt. Ltd., India and the sequence was used for BLAST search against the non-redundant database.

### *In silico* analysis of *AcPDS*

The exon–intron pattern of the *AcPDS* gene of cv. B. Super was analyzed using Gene Structure Display Server 2.0 (http://gsds.gao-lab.org/). The physicochemical properties, such as molecular weight and isoelectric point (pI), were calculated using the protparam tool (http://web.expasy.org/protparam/). The online Pfam tool (https://pfam.xfam.org/) was used to detect the presence of a conserved domain in the AcPDS.

### Multiple sequence alignments and phylogenetic tree reconstruction

The protein sequences of AcPDS of onion cv. B. Super and PDS from 18 different plant species from monocots and dicots were aligned using the MUSCLE program, and the aligned file was subjected to phylogenetic tree analysis. A phylogenetic tree was reconstructed in MEGA 11 (Tamura et al., 2021) using the maximum likelihood method, the Jones–Taylor–Thornton (JTT) model with a 1,000 bootstrap value.

### Selection of targets and construction of CRISPR/Cas9 vectors

Based on onion cv. B. Super *AcPDS* gene sequences, we targeted exon 3 and exon 4 regions of the *AcPDS* gene for CRISPR/Cas9-based genome editing, as they are located in the N-terminal region of the conserved amino-oxidase domain. Two different guide RNAs targeting exon 3 (*AcPDS* exon 3 gRNA-GAAGCTAGAGATGTTCTGGGAGG) and exon 4 (*AcPDS* exon 4 gRNA-GGTTGCTGCTTGGAAAGACAAGG) were predicted on the positive strand by the CRISPR-P2.0 (http://crispr.hzau.edu.cn/) web-based tool with highest efficacy. To elucidate the conserved sequence of target regions, a primer set was designed in the flanking regions spanning both target sites and used for PCR amplification of the *AcPDS* fragment using genomic DNA from 20 individual plants of onion cv. B. Super as a template and as per the PCR conditions described in **Supplementary Table S1**. After confirming the conserved target sequences, *Bsa*I adaptors (5′GGCA in the top strand and 5′AAAC in the bottom strand) were added to the predicted gRNAs, and oligomers of both gRNAs were synthesized. These oligomers with *Bsa*I overhangs were duplexed and cloned independently at the *Bsa*I site of the CRISPR/Cas9-based binary vector pRGEB31, under the control of the OsU3 promoter and *Pol*III terminator. The insertion and stability of the final binary vector constructs were confirmed by Sanger sequencing. The resulting constructs were named as pRGEB31-AcPDS E3 target and pRGEB31-AcPDS E4 target. Both binary vectors were independently introduced into the *Agrobacterium tumefaciens* strain LBA4404 by employing the tri-parental mating method.

### *Agrobacterium*-mediated transformation of onion

*Agrobacterium* LBA4404 harboring *AcPDS* gene editing constructs, *viz.* pRGEB31-AcPDS E3 target and the pRGEB31-AcPDS E4 target separately were used for co-transformation. A total of 617 eight-week-old embryogenic calli of onion were co-transformed using *Agrobacterium*-mediated transformation as described by Manape et al. (2022) in seven independent batches. An *Agrobacterium*-infected calli was blot-dried on sterile filter paper, transferred to the co-cultivation medium for 3 days, and then transferred to the resting medium for 15 days. Later, these calli were directly transferred to a shooting medium (devoid of hygromycin B) and subcultured regularly on the fresh shooting medium at intervals of 15 days. After shooting induction, the regenerated shoots were transferred to the rooting medium (½ strength Murashige and Skoog medium) to develop into complete plantlets. In each batch, 30–34 calli were also maintained without co-cultivation as an experimental control and subcultured routinely as per co-cultivated calli.

### Identification and sequence analysis of putatively transformed regenerated shoots

The morphology of the regenerated shoots from co-cultivated calli on the shooting medium was visually observed 8-weeks after co-cultivation. The genomic DNA was extracted from phenotypically distinct putatively transformed shoots and non-transformed shoots using the GenElute Plant Genomic DNA Miniprep Kit (Sigma) as per the manufacturer’s instructions. To investigate the presence of the *Cas9* gene in phenotypically distinct shoots, the Cas9 F/Cas9 R primer set was used for amplification of the internal sequence of the *Cas9* gene using 100 ng of DNA as a template as per the PCR conditions described in **Supplementary Table S1**. Whereas, to investigate the mutation induced in the exon 3 or exon 4 regions of *AcPDS* in the phenotypically distinct calli, PCR amplification of the *AcPDS* fragment harboring both target sites was carried out by using the DNA of these calli as a template and PrimeSTAR^®^ GXL DNA Polymerase (DSS Takara) with the help of another primer set (*AcPDS*g F/*AcPDS*g R). PCR-amplified products from *Cas9*-positive shoots were cloned into the pJET1.2 vector using a CloneJET PCR Cloning Kit (ThermoFisher Scientific), and 10 clones of each were sequenced using Sanger’s platform at Eurofins Genomics India Pvt. Ltd. The sequences of amplified fragments were aligned using the Clustal Omega online tool (https://www.ebi.ac.uk/Tools/msa/clustalo/). The identified InDels and substitutions were further validated by analyzing chromatograms using the Synthego ICE analysis online tool (https://ice.synthego.com/). In addition, we used purified PCR products, each with an equal concentration of 100 ng, from Cas9-positive plants. These products were amplified using flanking primers and then pooled together for sequencing using the Illumina Novaseq6000 platform to validate the results from Sanger sequencing. The raw reads were analyzed using the Cas-analyzer tool (Park et al., 2017). To estimate the frequency and type of mutation induced by each target, we used a 65-bp comparison range around the target CRISPR RNA (crRNA) sequences.

### Chlorophyll estimation

Total chlorophyll was extracted from 30 mg of shoot tissues from phenotypically distinct regenerated shoots and nontransformed green shoots as controls by following the protocol described by Arnon (1949). The chlorophyll content was estimated by the SPECTROstar Nano microplate reader (BMG Labtech, Germany) by recording the absorbance at 645 nm and 663 nm. Total chlorophyll content was estimated by using Arnon’s equation.


Chlorophyll a (µg/mg) = 12.7 (A663) - 2.69 (A645)

Chlorophyll b (µg/mg) = 22.9 (A645) − 4.68 (A663)

Total chlorophyll (µg/mg) = 20.2 (A645) + 8.02 (A663)

### Statistical analysis

The chlorophyll estimation experiment was carried out in triplicates, and the results of phenotypically distinct regenerated shoots were compared with control non-transformed green shoots. The average reduction in the chlorophyll content was calculated in percentage based on a mean value of distinct phenotype compared with the control regenerated shoots. The data were presented in the mean ± SD format and analyzed by one-way ANOVA. The statistical significance was checked at *p* ≤ 0.05–0.001 (^*^ *p* ≤ 0.001) with respect to control. The statistical analysis was performed using Analysis ToolPak in Excel (2021).

## Results

### Identification and analysis of the *AcPDS* sequence

**Figure 1:**
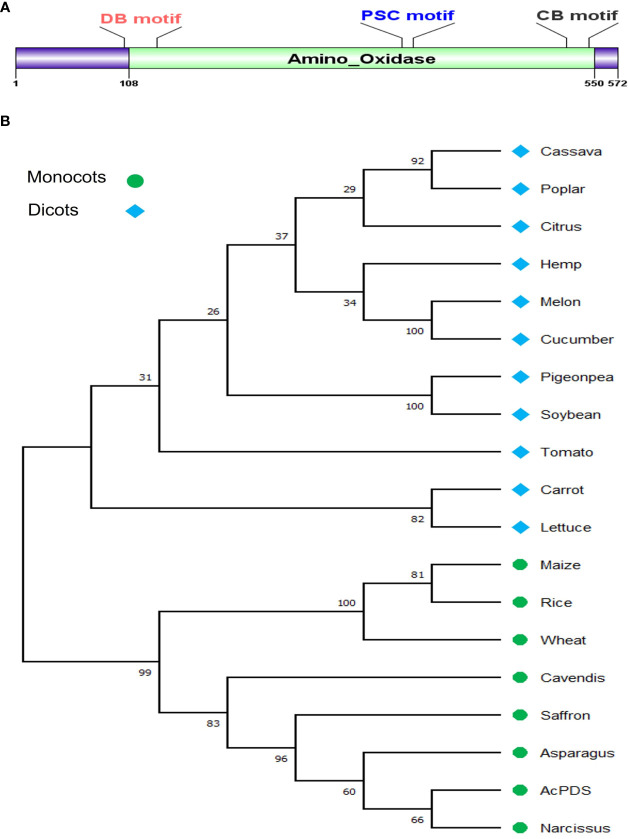
*In silico* analysis of AcPDS gene, **(A)** Schematic representation of conserved amino oxidase domain in AcPDS protein. **(B)** Phylogenetic relationship of AcPDS protein of *Allium cepa* L. cv. B. Super with PDS protein sequences from 11 dicot and seven monocot species was constructed using the MEGA 11 tool.

The nucleotide sequence of *Asparagus officinalis PDS* (XP_020273319.1) was used as a query to search for similar sequences in the draft genome sequence of onion (*Allium cepa* L.), and a single copy of *AcPDS* on chromosome 4 was identified. The coding sequence (CDS) of size 4,278 bp was amplified by PCR from the genomic DNA of onion cv. B. Super and submitted to the NCBI database (Accn. No. OP004915). The BLASTn analysis of the *AcPDS* of onion cv. B. Super revealed 23 mismatches and 4 gaps when compared to the draft genome sequence of the double haploid onion line DHCU066619 *PDS* (GBRN01009640.1) (https://www.oniongenome.wur.nl/). The sequence was confirmed by independent sequencing three times and was later predicted to be translated into a functional protein. The genomic *AcPDS* gene sequence of 4,278 bp size comprises 14 exons and 13 introns and has an open reading frame of 1,719 bp that encodes a protein of 572 amino acids with a predicted molecular weight of 63.9 kDa. Pfam database analysis of the AcPDS protein has shown the presence of a conserved amino-oxidase domain of the PDS family (**Figure 1A**). The *AcPDS* sequence showed 85.17% and 87.32% identity with the annotated *PDS* of *Narcissus* (AFH53812.1) and *Asparagus* (XP_020273319.1), respectively, suggesting a conserved function in the onion. The multiple sequence alignment analysis of the AcPDS protein had shown three characteristic conserved motifs of the PDS family, *viz*., the dinucleotide binding motif, the putative carrier motif, and the carotenoid-binding motif responsible for the catalytic activity of AcPDS (**Supplementary Figure S1**). The phylogenetic analysis of the AcPDS protein along with the PDS proteins from 18 different monocot and eudicot species showed that AcPDS clustered with the PDS proteins from monocot species and formed a separate clade, whereas PDS from dicot species formed a clade distinct from monocot plants (**Figure 1B**).

### Transformation of onion with CRISPR/Cas9 constructs and evaluation of phenotype

**Figure 2:**
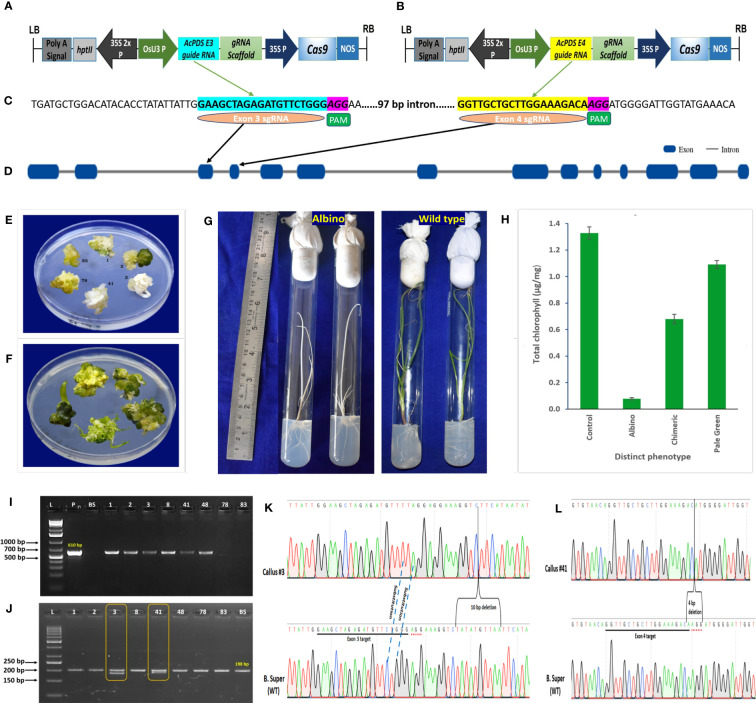
CRISPR/Cas9-mediated genome-editing of onion by targeting *phytoene desaturase* (*AcPDS*) gene. **(A)** Schematic presentation of binary vector pRGEB31-AcPDS E3 target. **(B)** Schematic presentation of binary vector pRGEB31-AcPDS E4 target. **(C)** Protospacer sequences for exon 3 target (cyan highlighted) and for exon 4 target (yellow highlighted); PAM sequences in pink highlighted. **(D)** Schematic representation of gene structure of AcPDS isolated from onion cv. B. Super, showing the gRNA targets. Blue blocks indicate exons of the gene. **(E)** Co-cultivated regenerated shoots showing three distinct phenotypes, *viz.* albino (regenerated shoots #3 and #41), chimeric (regenerated shoots #1 and #2), and pale green (regenerated shoots #78 and #83). **(F)** Nontransformed control regenerated shoots showing dark green shoots. **(G)** Regenerated plantlets showing albino and normal green wild-type phenotypes. **(H)** Chlorophyll content estimation in the three phenotypically distinct regenerated shoots. Bars represent the mean ± SD of the three independent replicates (*n* = 3). **(I)** Representative PCR analysis of phenotypically distinct shoots to confirm the presence of *Cas9* gene. **(J)** PCR analysis of albino and chimeric shoots showing varying sizes of amplicons in the target regions. (L, 1 kb plus ladder in **(H)** and 50 bp ladder in **(I)**; P, pRGEB31-plasmid (positive control); BS: onion cv. B. Super (negative control); 1, 2, 3, 8, 41, 48, 78, and 83: phenotypically distinct co-cultivated regenerated shoots). **(K, L)** Schematic chromatogram presentation of site-specific mutations of *AcPDS* induced by exon 3 and exon 4 sgRNAs in albino shoots regenerated from callus #3 and #41, respectively. Dot lines specify the type of mutation, and parentheses indicates large (4 nt and 10 nt) deletions. PAM is in red dots, and protospacers are underlined.

A total of 617 embryogenic calli were co-transformed with the pRGEB31-Ac*PDS* E3 target (**Figure 2A**), harboring exon 3 sgRNA (**Figure 2C**), and the pRGEB31-Ac*PDS* E4 target (**Figure 2B**), harboring exon 4 sgRNA (**Figure 2D**), using *Agrobacterium*-mediated transformation in seven independent batches. As mutations in the *PDS* gene resulted in visible albino or chimeric phenotypes, the calli were not screened on selection steps (MS medium supplemented with 50 mg/L of hygromycin B) of Manape et al. (2022) to reduce the time of regeneration protocol and were directly regenerated on the shooting medium devoid of hygromycin B. After 8-weeks of co-cultivation, the co-cultivated and non-transformed calli had regenerated into shoots. Shoots regenerated from non-transformed calli with shooting efficiency of 76%, and all were normal green (**Figure 2F**). On the contrary, out of 617 co-transformed calli, 456 shoots were regenerated, of which 21 shoots had three additional different phenotypes along with the normal green phenotype, *viz*., 9.52% complete albino (two of 21; callus #3 and #41), 19.04% chimeric (four of 21; callus #1, #2, #8, and #48), and 71.42% pale green (15/21) (**Figure 2E**). The regenerated, phenotypically distinct shoots were rooted on rooting media. On rooting media, the pale green shoots were turned into normal green plantlets, whereas chimeric shoots were dried off quickly, and newly emerged leaves were not chimeric and looked more like leaves of the wild-type. The albino plants retained the albino phenotype throughout the life cycle but showed a slower growth rate than non-transformed control plants (**Figure 2G**).

The chlorophyll content also varied in these different phenotypes, viz. 0.073 ± 0.008 μg/mg to 0.085 ± 0.012 μg/mg in albino, 0.644 ± 0.071 μg/mg to 0.724 ± 0.033 μg/mg in chimeric, and 1.027 ± 0.026 μg/mg to 1.187 ± 0.019 μg/mg in the pale green phenotypes, whereas the chlorophyll content in the non-transformed control regenerated shoots was 1.328 ± 0.046 μg/mg (**Figure 2H**). There was significant reduction of the chlorophyll content in the albino (94.07%) and chimeric (48.79%) and a nonsignificant reduction in the pale green (17.80%) shoots as compared to the control non-transformed green shoots.

### Characterization of the editing in the *AcPDS* gene

The PCR analysis of these 21 phenotypically different shoots showed the presence of a 610-bp-sized *Cas9* gene fragment in six regenerated shoots (**Figure 2I**). Out of six, two had complete albino and four had chimeric (mixture of albino and green) shoot morphology, whereas the *Cas9* gene was absent in the pale green transformed and non-transformed control regenerated shoots. With the help of the *AcPDS*g F/*AcPDS*g R primer set, DNA fragments of size 198 bp spanning both the target sites were PCR amplified from these six transgenic shoots and from non-transgenic control shoots. Moreover, an additional 188 bp and 194 bp fragments were amplified in albino shoots #3 and #41, respectively (**Figure 2J**). The sequence alignment of 188 bp and 194 bp fragments with the reference sequence of onion cv. B. Super revealed that shoot #3 had 2 nucleotide substitutions in the exon 3 target site along with 10 nucleotide deletions (+8 to +17 bases from exon 3 PAM site) in the downstream region of the exon 3 target site (**Figure 2K**). Shoot #41 showed a 4-bp deletion within the exon 4 target site, including the PAM sequence (**Figure 2L**).

A comparative analysis was conducted using the Synthego ICE analysis tool on the Sanger view plot of the wild-type B. Super chromatogram, alongside the chromatograms of shoots #3 and #41, to validate multiple sequence alignment results. Synthego ICE tool analysis of shoot #3 revealed the cut site in the exon 3 gRNA target sequence along with 2 bp substitutions, and no cut site was predicted in the exon 4 gRNA sequence. Additionally, a 10-bp deletion was observed in the downstream region of the exon 3 PAM site of shoot #3, accounting for 30% of the total InDel mixture (**Supplementary Figure S2B**). On the other hand, the analysis of shoot #41 using the Synthego ICE tool revealed the cut site in the exon 4 gRNA target sequence, while no cut site was predicted in the exon 3 gRNA target site. An InDel plot of shoot #41 demonstrated a 4 bp deletion within the exon 4 PAM site, constituting 58% of the total InDel mixture (**Supplementary Figure S3B**). These findings clearly indicated that the observed InDels in shoots #3 and #41 resulted from editing due to the action of exon 3 and exon 4 sgRNAs, respectively.

The chimeric transgenic onion shoots did not show any editing in and around both target sites and had a similar sequence as that of the wild-type. This could be due to the limited number of clones (i.e., 10 clones per sample) were sequenced by Sanger sequencing. Thus, the genome editing efficiency of 33.33% (two of six) was observed among the PCR-positive regenerated onion shoots.

### Deep sequencing analysis

We performed deep amplicon sequencing of the *AcPDS* target region on six *Cas9*-positive shoots using the Illumina NovaSeq6000 platform with 2 × 150 bp chemistry. This yielded 5.96 million paired-end reads, corresponding to 2.54 Gb of sequence data. Using the Cas-analyzer tool, we analyzed the *AcPDS* Illumina amplicons and identified insertions, deletions, and substitution-type mutations. Our next-generation sequencing (NGS) data indicated InDel frequencies of 63.4% and 1.237% in and around exon 3 and exon 4 sgRNAs, respectively (**Table 1**). Among the 63.4% InDel frequency of exon 3 sgRNA, 60.21% were deletions, primarily of size 10 bp downstream to the target site of exon 3 sgRNA (58.75%), while 3.19% were insertions, mostly of size 3 bp and 8 bp downstream to the target site of exon 3 sgRNA (2.86%) (**Supplement S2**; **Supplementary Figure S4**). We also observed 3.04% substitutions, of which 0.22% were at the exon 3 sgRNA target, featuring specific base changes (i.e., C is replaced by T at the 16^th^ position and G is replaced by A at the 18^th^ position of exon 3 gRNA). These same substitutions also appeared in deletion counts (**Supplementary Figure S6**). After combining these counts manually, the substitution frequency for these specific bases in exon 3 sgRNA increased from 0.22% to 54.84%. The remaining fraction of InDels (1.46% deletions and 0.33% insertions of 1 to 2 bp) and substitutions at various positions (2.8%) were around the exon 3 sgRNA, possibly from chimeric shoots. As deep sequencing could be able to detect the mutants other than editing identified by the Sanger platform, therefore, these edits could be possibly derived from chimeric shoots. Regarding the exon 4 target, the overall InDel frequency was 1.237%, primarily deletions of size 4 bp at the PAM site (1.1%), including the last nucleotide of exon 4 sgRNA, with negligible insertion frequency (**Supplement S3**; **Supplementary Figure S5**). We also observed negligible (0.54%) substitutions in exon 4 sgRNA. Overall, our NGS data were consistent with Sanger sequencing data. Additionally, we detected low-frequency InDels and substitutions in the NGS data, potentially from chimeric shoots (**Supplement S2**, **S3**).

**Table 1:** Analysis of NGS data.

Target	Total sequences	Pooled and assembled sequences with more than minimum frequency	Insertions	Deletions	InDels	Substitutions
Exon 3 sgRNA	5,959,762	2,953,153	94,163 (3.19%)	1,778,061 (60.21%)	1,872,224 (63.4%)	83,539 (2.83%)
Exon 4 sgRNA	5,959,762	1,108,502	83 (0.007%)	13,626 (1.23%)	13,709 (1.237%)	6,007 (0.54%)

The number in parentheses indicates frequency in percentage.

## Discussion

The CRISPR/Cas9 technology has so far been used in numerous plant species for basic research and trait development. Apart from the evaluation of gene functions, the application of CRISPR/Cas9 technology has great potential in onion for manipulation of a metabolic pathway to alter flavor profile, flowering behavior, quality, and traits of agronomic importance for increasing yield potential. Though onion is one of the most important vegetable crops throughout the world, the study for CRISPR/Cas9-mediated gene editing in onion has not been conducted yet. To the best of our knowledge, the present study is the first report on CRISPR/Cas9-mediated genome editing in onion.

The *PDS* gene is involved in the carotenoid biosynthesis pathway, and its loss-of-function mutation leads to the development of the photobleached or albino phenotype, which can be easily and quickly detected by naked eye and does not require any biochemical, physiological, or microscopic assays. Therefore, *PDS* has been widely used as a common visual marker to validate the practicability of genome editing by CRISPR/Cas9 in several plant species, *viz. populus* (*Populus angustifolia*) (Fan et al., 2015), apple (*Malus prunifoli*) (Nishitani et al., 2016), grape (*Vitis vinifera*) (Nakajima et al., 2017), cassava (*Manihot esculenta*) (Odipio et al., 2017), banana cv. Rasthali (*Musa acuminata* cv. Rasthali) (Kaur et al., 2018), cavendish banana (*Musa acuminata* subgroup Cavendish) (Naim et al., 2018), melon (*Cucumis melo*) (Hooghvorst et al., 2019), strawberry (*Fragaria ananassa*) (Wilson et al., 2019), lettuce (*Lactuca sativa*) (Henderson et al., 2020), poplar (*Populus tomentosa Carr*) (Fan et al., 2015; Wang et al., 2020), and yam (*Dioscorea rotunda*) (Syombua et al., 2021). Thus, we targeted the *AcPDS* gene to evaluate the editing efficiency induced by the CRISPR/Cas9 system.

A detailed analysis of the targeted gene and its putative functions is a prerequisite for any genome editing studies. We retrieved the *AcPDS* sequence from the draft onion genome based on a homology search. As the B. Super cultivar was used for the genome editing study, the gene was additionally isolated from the Indian onion cv. B. Super and afterward employed for designing and constructing targets for genome editing studies. *PDS* has been reported as a single copy gene in many crop species like melon (Hooghvorst et al., 2019), cassava (Odipio et al., 2017), and cavendish banana (Naim et al., 2018). Likewise, BLAST*n* analysis also showed a single copy of the *PDS* gene in the draft genome sequence of onion. Furthermore, *in-silico* analysis of the AcPDS protein sequence from B. Super cultivar showed that it contained the characteristic signatures of the conserved amino-oxidase domain known from other crops (Li et al., 2013b; Kaur et al., 2017). Therefore, we targeted the 5′ region of a conserved amino-oxidase domain of *AcPDS* to ensure functional knockdown upon mutation. Moreover, we selected two gRNA targets in the amino-oxidase domain, i.e., in exon 3 and exon 4, to improve our chances of success and to evaluate any large deletions in addition to InDels, as they were used in other crops (Odipio et al., 2017; Ntui et al., 2020; Lu and Tian, 2022).

Onion is recalcitrant to transformation, has very low transformation efficiency (i.e., 0.2% to 2.5%), and transformation is highly genotype-dependent (Eady et al., 2000; Zheng et al., 2001; Eady et al., 2003; Zheng et al., 2005; Kamata et al., 2011; Naini et al., 2019; Manape et al., 2022). There are two reported techniques for the genetic transformation of onions: i.e., biolistic transformation and *Agrobacterium*-mediated transformation. A biolistic method is challenging to carry out within crop plants and requires expensive consumables such as gold particles or specialized machinery (e.g., gene gun). On the other hand, *Agrobacterium*-mediated is inexpensive, does not require any specialized equipment, and has been standardized in our laboratory for the onion cv. B. Super with 1% transformation efficiency (Manape et al., 2022). Therefore, we employed the same protocol to study the CRISPR/Cas9-based genome editing in the same onion cultivar and regenerated six *Cas9* PCR-positive shoots out of 617 co-cultivated calli with a comparable transformation efficiency of 0.97%.

In general, putatively transformed calli are selected on growth medium by using either negative agents, namely, kanamycin, hygromycin B, and spectinomycin, or positive ones like mannose (Bibi et al., 2013; Rastogi et al., 2018). Negative selection can inhibit and delay shoot growth, cause chlorosis in shoots, and produce pale green phenotypes, as observed in crops like sugarcane (Rastogi et al., 2018) and cotton (Bibi et al., 2013). The pale green phenotypes were also observed due to Cas9-induced InDels in the *PDS* gene of the Cavendish banana (Naim et al., 2018) and hybrid poplar (Wang et al., 2020). This necessitates the screening of pale green shoots to differentiate between non-transformed and transformed shoots, which is a laborious and time-consuming process. In the onion variety B. Super, embryogenic calli induce shoots within 3 weeks on a shooting medium devoid of hygromycin B, whereas transformed calli take 6–8 weeks in the presence of hygromycin B, occasionally producing shoots with chlorotic effects (unpublished data). However, using a positive selection method requires optimization of the selection agent concentration, which is also labor- and time-intensive. Given that the regeneration protocol was already standardized for onion cv. B. Super, we transferred co-cultivated calli directly to a shooting medium without hygromycin B. This approach eliminated confusion over pale green shoots and expedited the regeneration protocol for quick validation of CRISPR/Cas9-mediated genome editing. Our study successfully regenerated 21 shoots with varying degrees of albino or chlorophyll loss within 7–8 weeks using *Agrobacterium*-mediated co-transformation of embryogenic calli of onion with two independent *AcPDS* targeting CRISPR/Cas9 constructs. This is significantly faster than the 22–24 weeks required by the transformation protocol that we had developed earlier (Manape et al., 2022). Of these 21 shoots, two were complete albinos, four were chimeric, and 15 were pale green. Similar phenotypes have been reported in *PDS* knockout experiments in various plant species (Pan et al., 2016; Naim et al., 2018; Hooghvorst et al., 2019; Henderson et al., 2020; Wang et al., 2020; Lu and Tian, 2022).

To ascertain genome editing in onion, PCR analysis was performed with a flanking primer set in six *Cas9*-positive regenerated shoots, and multiple clones of each were sequenced on Sanger’s platform to investigate mutations within and around one or both target sites. Sanger sequencing results revealed InDels and substitutions in and around the target sites of both sgRNAs in the albino phenotypes. A similar type of mutation has also been reported in other plants, including *Arabidopsis (Arabidopsis thaliana)* (Feng et al., 2014), apple (*Malus prunifoli*) (Nishitani et al., 2016), and Rasthali banana (*Musa acuminata* cv. Rasthali) (Kaur et al., 2018). The InDels recorded here could have resulted from a nonhomologous end-joining repair (NHEJ) pathway following the integration of T-DNA (Fan et al., 2015; Nishitani et al., 2016).

InDels are the most common mutations in CRISPR/Cas9-mediated gene editing, induced by NHEJ. The evaluation of the Sanger sequencing data, using multiple sequence alignment and the Synthego ICE Analysis tool, was found to be consistent with the results from NGS data. Both sets of data indicated similar InDels in the vicinity of the target sgRNAs. NGS data showed a high level (63.4% in exon 3 sgRNA) to a low level (1.1% in exon 4 sgRNA) of InDel frequencies. This clearly demonstrated a significant difference in the editing efficiency of exon 3 and exon 4 sgRNAs. The GC content of the target sgRNA sequence and secondary structure of targeted sgRNAs affect the editing efficiency of Cas9 (Wang et al., 2014; Zhang et al., 2014; Ma et al., 2015). Both the sgRNAs had 50% GC content. Therefore, we analyzed the secondary structures of both target sgRNAs. Analyzing the secondary structure revealed that exon 3 target sgRNA had pairings of only 2 to 4 bp (i.e., continuous pairing less than 5 bp) in the target sgRNA sequence. However, exon 4 target sgRNA had a typical stem-loop structure with a pairing of continuous 7 and 4 bp of the target sgRNA sequence (**Supplementary Figure S7**). Secondary structures of target-sgRNAs with more than continuous 6 bp pairing of the target sgRNA sequence with stem-loop structures had significantly low editing efficiency, possibly due to inhibition of binding of the sgRNA to the target strand attributed to the formation of stem-loop structures (Ma et al., 2015).

Along with InDels, we also found a high frequency (54.84%) of substitutions in the exon 3 sgRNA sequence. Similar findings on the high level of substitutions mediated by CRISPR/Cas9 have been made by other studies. For example, in cotton (*Gossypium hirsutum* L.) protoplasts, only substitutions were found (Chen et al., 2017); in melon protoplasts and plants, 91% of mutations were substitutions (Hooghvorst et al., 2019); in cassava plants, substitutions occurred more frequently than InDel mutations (Odipio et al., 2017); in soybean (*Glycine max* L.) protoplasts and plants, a high level of substitutions were reported in the Cas9-edited events (Sun et al., 2015); and in rice (*Oryza sativa* L.), 25%–45% of the examined mutations were substitutions (Macovei et al., 2018). Observation of nucleotide substitution downstream of the target region implies activation of the homologous recombination (HR) pathway to repair double-strand breaks created in *AcPDS*. Simultaneous triggering of HR and NHEJ repair pathways proceeding integration of CRISPR/Cas9 gene T-DNA was documented earlier in apple (*Malus prunifoli*), *Arabidopsis* (*Arabidopsis thaliana*), tobacco (*N. benthamiana*), cassava (*Manihot esculenta*), and cotton (*Gossypium hirsutum*) (Li et al., 2013a; Nishitani et al., 2016; Gao et al., 2017; Odipio et al., 2017).

Four regenerated chimeric shoots showed the presence of the *Cas9* gene, but no editing was detected in and around the target sites when Sanger sequencing results were analyzed. However, NGS data revealed additional InDels and substitutions, albeit at very low frequencies. As we sequenced only a few clones from each chimeric shoot using the Sanger platform, it is likely that we were not able to detect these very low frequencies of InDels and substitutions, which potentially could have been induced by transient expression of the Cas9 gene and sgRNA from the transformed construct. Bleached patches of tissue, similar to our observations, were reported in a study on yam, and they were attributed to the transient expression of Cas9 and PDS-targeted sgRNA (Syombua et al., 2021). While this is a possibility in our study, another plausible explanation could stem from the use of a non-selective medium leading to the regeneration of chimeric transformants.

In our study, out of the six *Cas9*-positive shoots identified, two demonstrated CRISPR/Cas9-mediated genome editing in the *AcPDS* gene, yielding an editing efficiency of 33.33%. Mutation efficiency in monocotyledons using 35S promoter-driven Cas9 constructs is 1.8%–81% (Basu et al., 2023). Therefore, mutation efficiency is moderate and can be improved in future studies. We used the pRGEB31 vector, which contains the cauliflower mosaic virus (CaMV35S) and *Oryza sativa* U3 promoter for driving the expression of *Cas9* and sgRNAs, respectively. Optimal CRISPR/Cas9 function requires high transcription levels of both the sgRNA and Cas9 proteins (Mao et al., 2016). Editing efficiency is significantly influenced by the choice of promoter driving *Cas9*, with endogenous constitutive promoters yielding high transgene expression and, consequently, higher mutation efficiencies (Hassan et al., 2021). Similarly, the endogenous U6 promoter has been shown to enhance sgRNA expression and thus the overall CRISPR/Cas9-mediated editing efficiency by up to 87% (Yan et al., 2015; Zhang et al., 2018). As the promoters used in our study were not endogenous, it may have contributed to the less effective performance of the CRISPR/Cas9 system. Therefore, the utilization of endogenous promoters for Cas9 and sgRNA expression could be a more effective strategy for boosting CRISPR/Cas9 efficiency in onions.

We estimated the chlorophyll content in the regenerated albino, chimeric, pale green, and non-transformed control shoots to investigate the impact of AcPDS mutations. We observed a significant reduction in chlorophyll content in albino and chimeric shoots. InDels produced frameshift mutations and ectopic stop codons in the *AcPDS* gene of albino shoots. These alterations led to the early termination of AcPDS protein, leading to albinism; consistent with findings in Rasthali banana (Kaur et al., 2018). The variegation and reduced chlorophyll content in chimeric phenotypes could be the result of a transient expression of Cas9-mediated alterations in *AcPDS*. Similar observations were reported in yam, where bleached patches on shoots were linked to *Agrobacterium*-mediated transient expression of Cas9 and *PDS* sgRNA (Syombua et al., 2021). The nonsignificant reduction in chlorophyll content in the 15 pale green calli may be attributed to somaclonal variation induced by frequent culturing on the shooting medium. Further detailed analysis is required to confirm this hypothesis. In summary, the substantial decrease in chlorophyll content in albino shoots indicates a functional knockout of the *AcPDS* gene. This disruption likely affects both the chlorophyll and gibberellic acid biosynthesis pathways. The slow growth rate observed in albino plants may be due to the disruption of gibberellic acid biosynthesis, which hinders their growth and development. This is supported by a study in *Arabidopsis*, where the application of exogenous GA_3_ partially reversed the dwarf phenotypes of *AcPDS* mutants (Qin et al., 2007).

In conclusion, the CRISPR/Cas9-mediated *AcPDS* knockout showcased in this study produced a readily observable mutant phenotype within a rapid timeframe of 8-weeks. This strategy provides a valuable tool for accelerating the evaluation and optimization of CRISPR/Cas9-mediated genome editing in onions, ultimately contributing to the improvement of their traits. While the observed editing efficiency in our study was moderate, the use of an endogenous promoter-driven Cas9 and sgRNA could potentially enhance it in future applications.

## Data availability statement

The data presented in the study are deposited in the NCBI database repository, accession number PRJNA976681 (http://www.ncbi.nlm.nih.gov/bioproject/976681). The original contributions presented in the study are included in the article/**Supplementary Material**. Further inquiries can be directed to the corresponding author.

## Author contributions

PM, TKM, and SA conceived and designed the experiments. PM and TKM conducted all the experiments. PM, TKM, and SA drafted the manuscript. SA and VS carried out the data analysis and manuscript editing. SA supervised the entire work. All authors contributed to the article and approved the submitted version.
