# Supplementary material for: CRISPR/Cas9-mediated editing of PHYTOENE DESATURASE gene in onion (Allium cepa L.)
Source: Front Plant Sci. 2023 Aug 28;14:1226911. doi: 10.3389/fpls.2023.1226911 (PMC10494252; doi:10.3389/fpls.2023.1226911)
Supplement: Supplementary file 3 [file DataSheet_3.docx]

**Supplementary Information**

**CRISPR/Cas9-mediated editing of *PHYTOENE DESATURASE* (*AcPDS*) gene in onion (*Allium cepa* L.)**

Pawan Mainkar^1†^, Tushar Kashinath Manape^1†^, Viswanathan Satheesh^2^ & Sivalingam Anandhan^1^*

*Corresponding author: [anandhans@gmail.com](mailto:anandhans@gmail.com)

^†^ These authors contributed equally to this work and share first authorship.


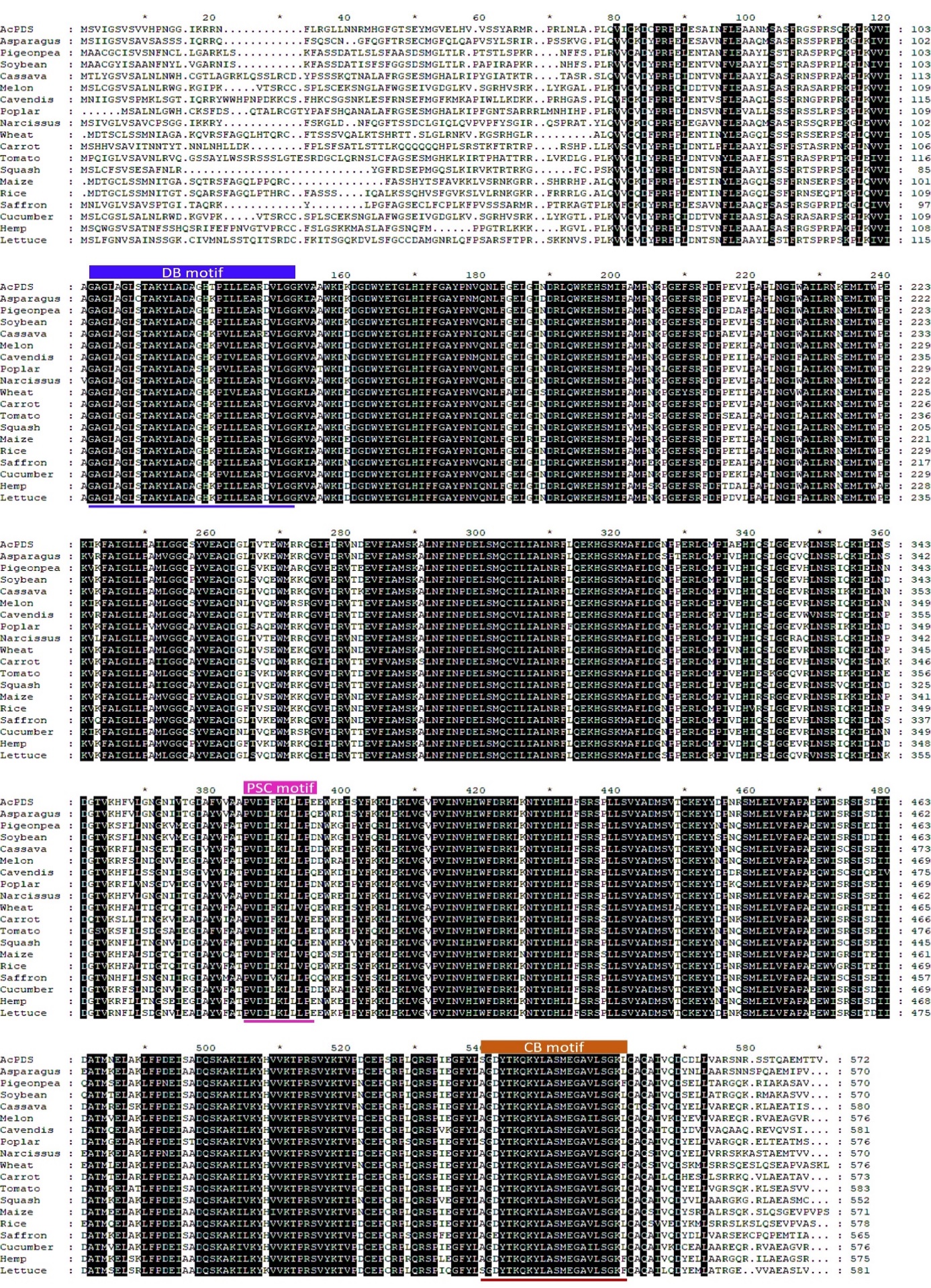


**Fig S1. Multiple sequence alignment of PDS protein from different plant species**

Blue highlighted sequence represents dinucleotide binding (DB) motif, pink highlighted represents putative carrier (PSC) motif and brown highlighted represents carotenoid-binding (CB) motif responsible for the catalytic activity of PDS, GenBank accession numbers of PDS used for multiple sequence alignment analysis are from monocots such as Onion(OP004915), Asparagus (XP_020273319.1), Cavendish (XP_008454070.1), Wheat (ACL36586.1), Maize (P49086.1), Rice (XP_015633101.1), Saffron (AAO24235.1), Narcissus (AFH53815.1) and from dicots like,Pigeonpea (XP_020213388.1), Soybean (NP_001236769.2), Cassava (XP_021613095.1), Melon (XP_008454070.1), Poplar (PNT51290.1), Carrot (NP_001316104.1), Tomato (NP_001234095.1), Citrus (AJT59422.1), Cucumber (XP_031740146.1), Hemp (QHD56482.1), Lettuce (XP_023766748.1)


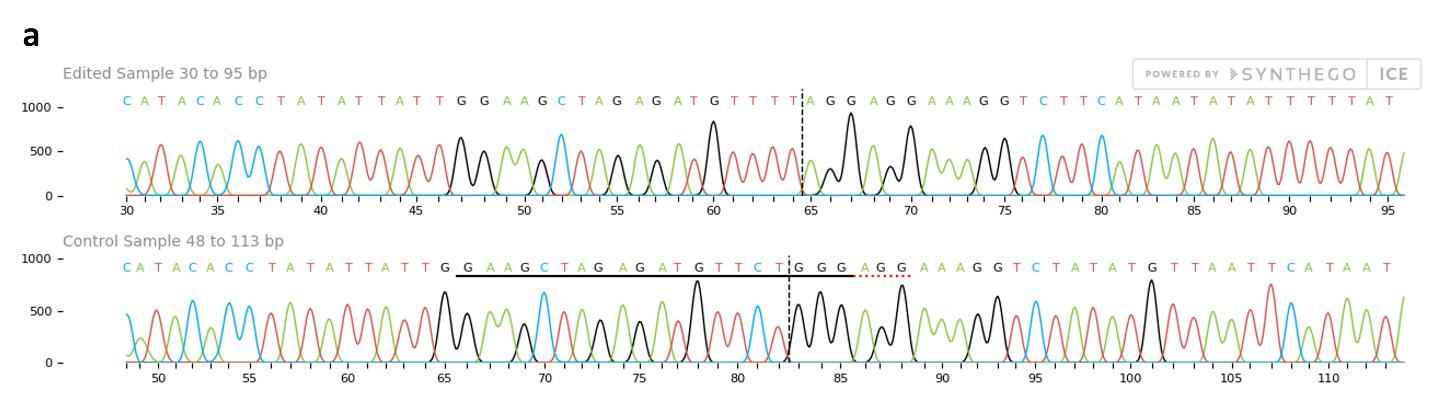


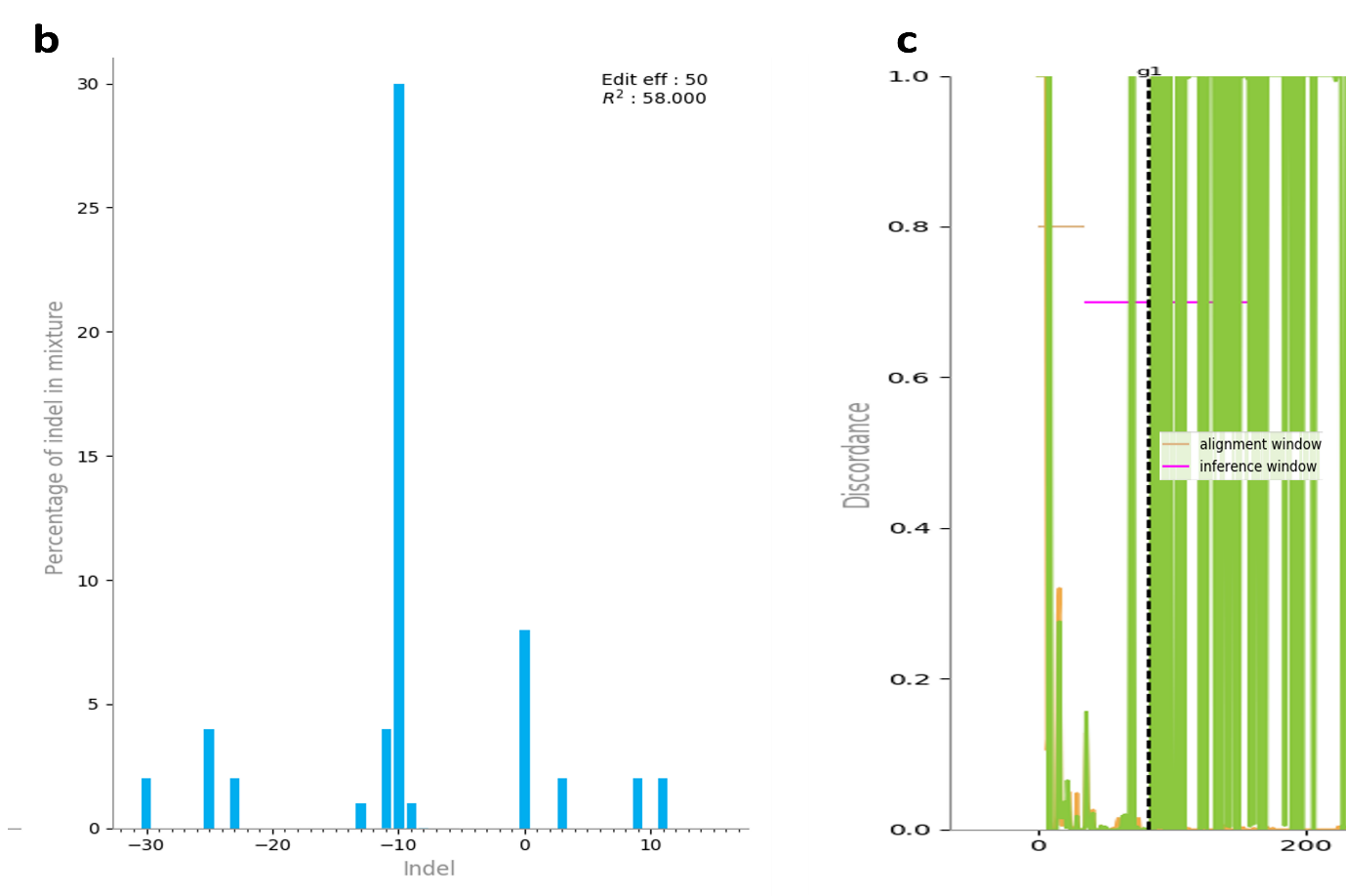


**Fig S2: Analysis of chromatogram of shoot #3**

The chromatograms of shoot #3 and wild-type B. Super (control) were compared using Synthego ICE Analysis online tool. **a.** Sanger sequence view plot showing edited region and mixed base calls sequences from both the control and the shoot #3 .ab1 files. The horizontal solid black and dotted red lines represent the guide sequence and PAM site, respectively. The vertical black dotted line represents the actual cut site. **b.** The Indel plot displays the inferred distribution of indels in the edited sequence of shoot #3. **c.** the discordance plot detailed the level of alignment per base between the wild type (control) and shoot #3 in the inference window (the region around the cut site), which refers to the average amount of signal that disagrees with the reference sequence derived from the control trace file. On the plot, the green and orange lines are close together before the cut site and remain far apart near the cut site due to a typical CRISPR edit resulting in a jump in the discordance near the cut site and representing a significant level of sequence discordance. The discordance plot was cropped at the 3' end to remove pJET vector sequences, retaining only the amplified *AcPDS* and 5' end vector sequences for better visualization.


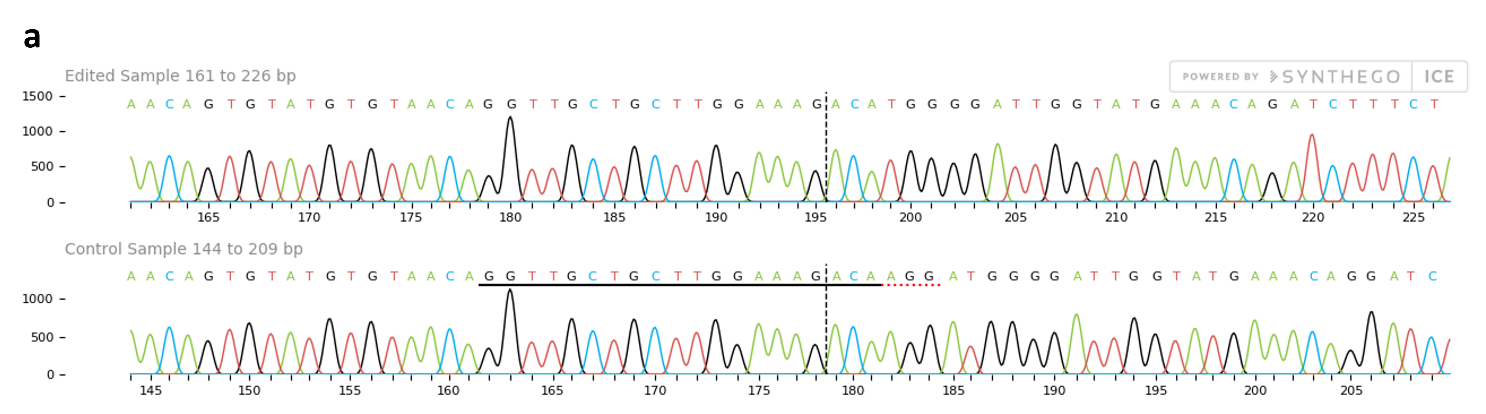


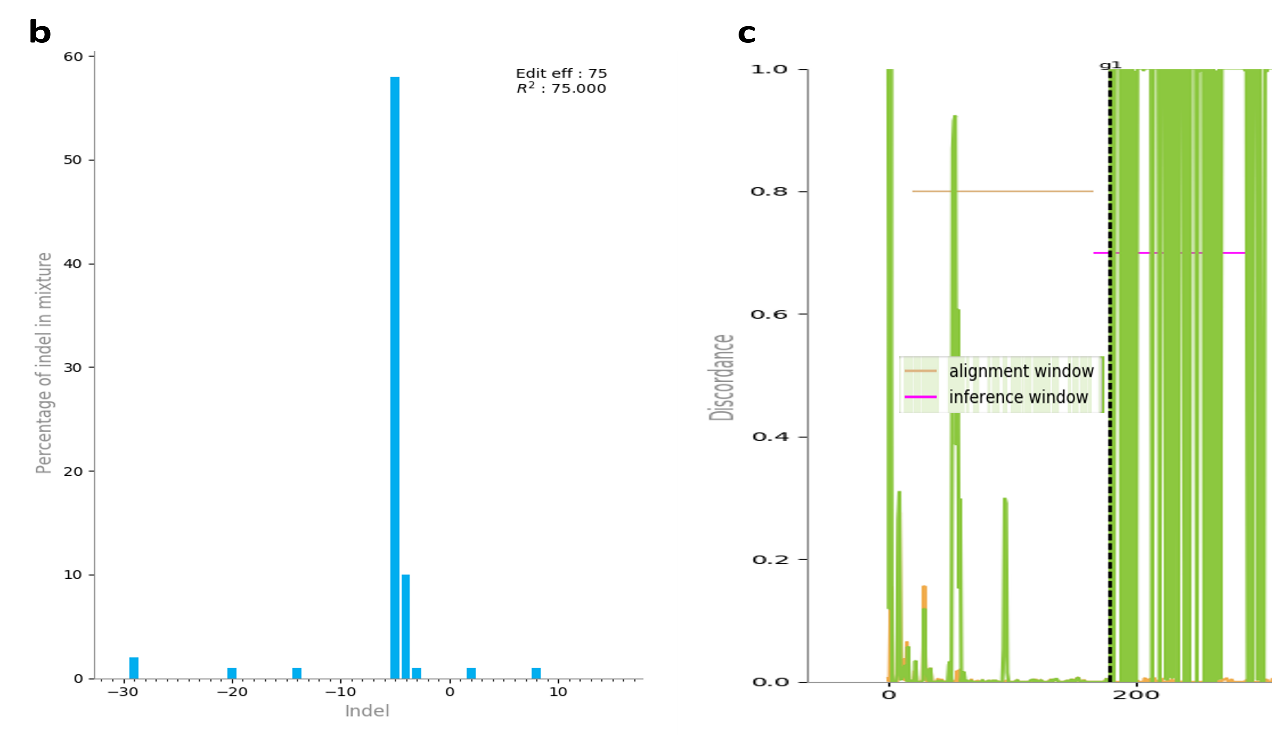


**Fig S3: Analysis of chromatogram of shoot #41**

The chromatograms of shoot #41 and wild-type B. Super (control) were compared using Synthego ICE Analysis online tool. **a.** Sanger sequence view plot showing edited region and mixed base calls sequences from both the control and the shoot #41 .ab1 files. The horizontal solid black and dotted red lines represent the guide sequence and PAM site, respectively. The vertical black dotted line represents the actual cut site. **b.** The Indel plot displays the inferred distribution of indels in the edited sequence of shoot #41. **c.** the discordance plot detailed the level of alignment per base between the wild type (control) and shoot #41 in the inference window (the region around the cut site), which refers to the average amount of signal that disagrees with the reference sequence derived from the control trace file. On the plot, the green and orange lines are close together before the cut site and remain far apart near the cut site due to a typical CRISPR edit resulting in a jump in the discordance near the cut site and representing a significant level of sequence discordance. The discordance plot was cropped at the 3' end to harboring pJET vector sequences, retaining only the amplified *AcPDS* and 5' end vector sequences for better visualization.


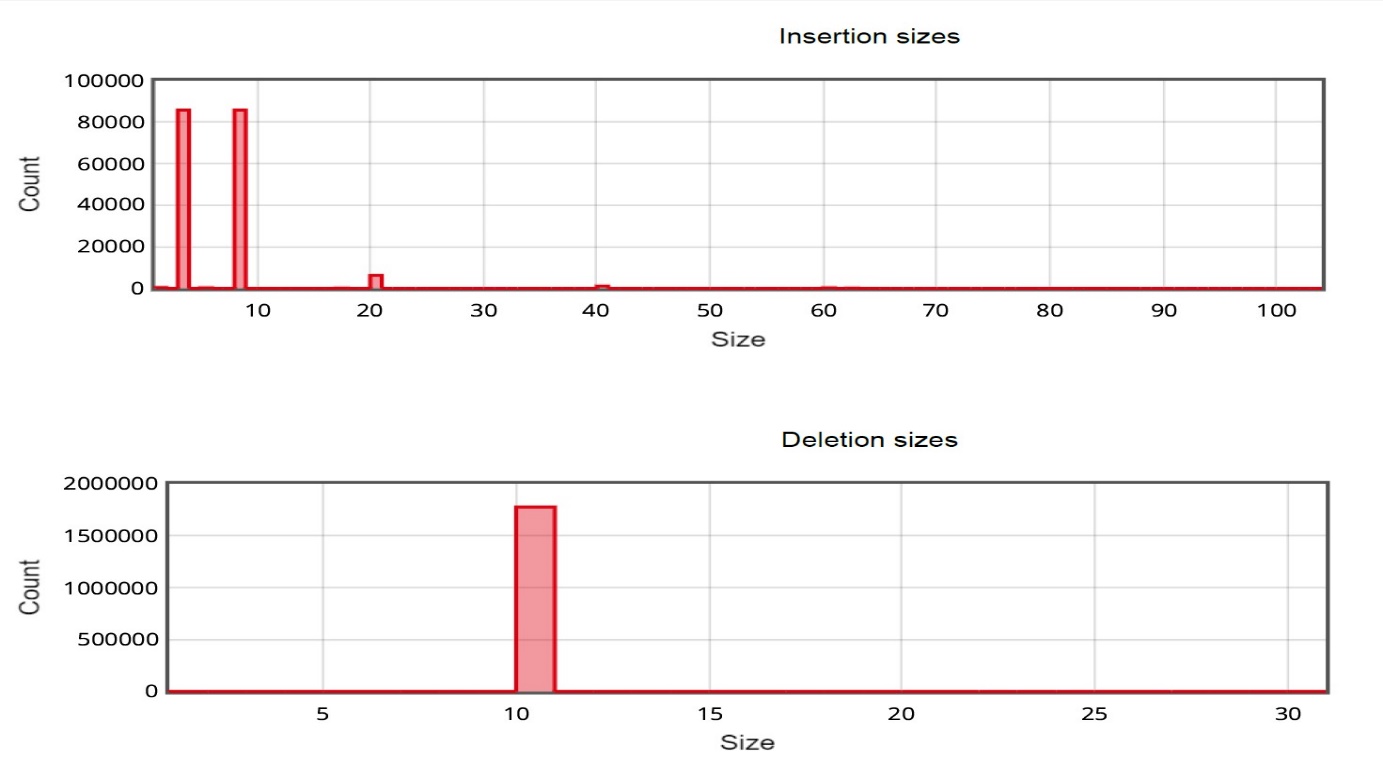

**Fig S4. Bar diagram depicting size and counts of Indels in and around exon 3 sgRNA**

The bar depicts number of InDel bp along with their count in the deep amplicon sequencing data


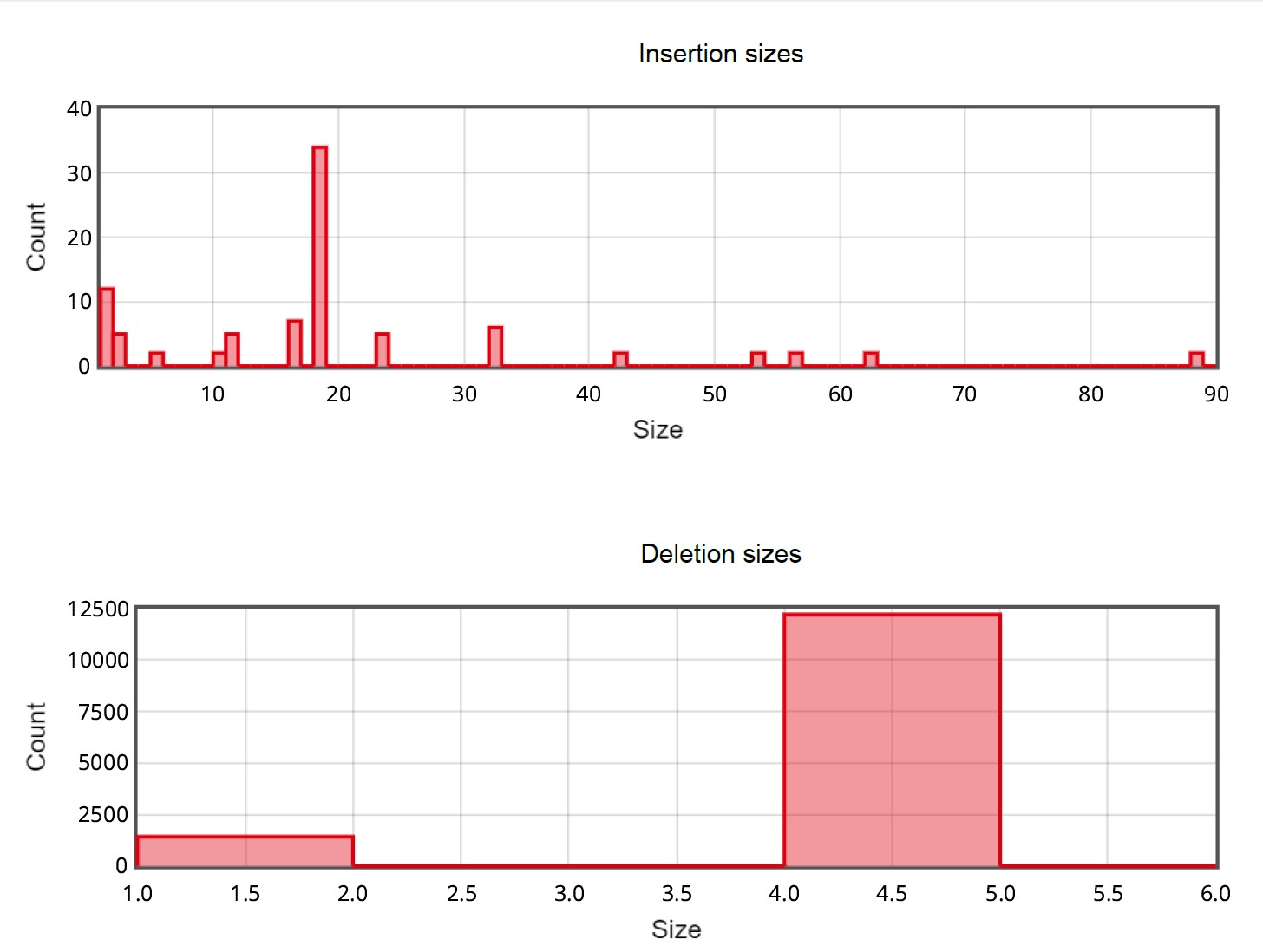


**Fig S5. Bar diagram depicting size and counts of Indels in and around exon 4 sgRNA**

The bar depicts number of InDel bp along with their count in the deep amplicon sequencing data


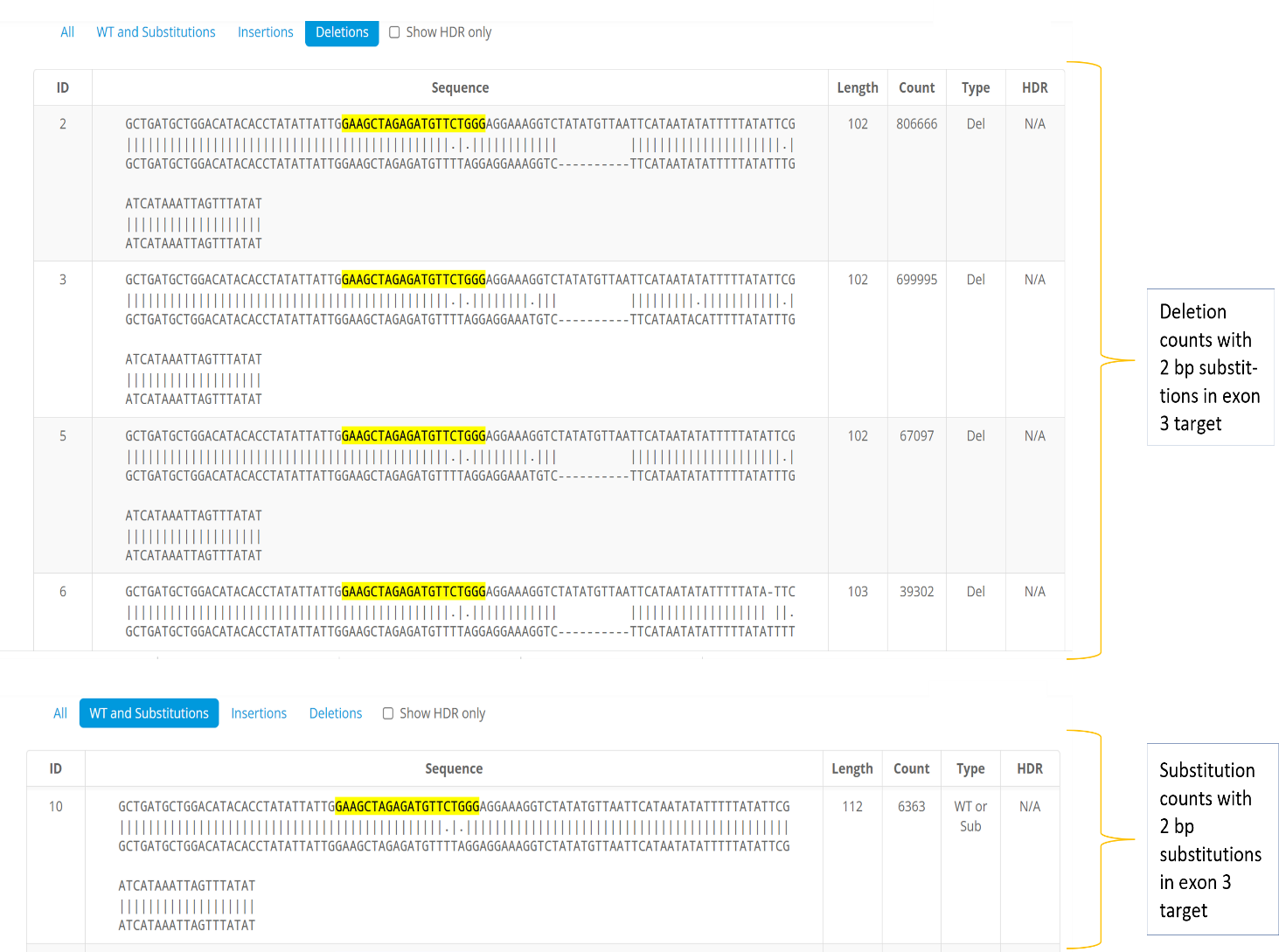


**Fig S6. Similar substitutions in deletion and substitution dataset**

Substitution of 2 bp (*i.e.* C is replaced by T at 16^th^ position and G is replaced by A at 18^th^ position) of exon 3 gRNA was reported in substitution frequency dataset. The similar substitutions were also detected in deletion frequency dataset. Thus, the substitution frequency for these two bases were calculated by combining their counts from substitution and deletion datasets, which resulted into increase in substitution frequency from 0.22% to 54.84%.


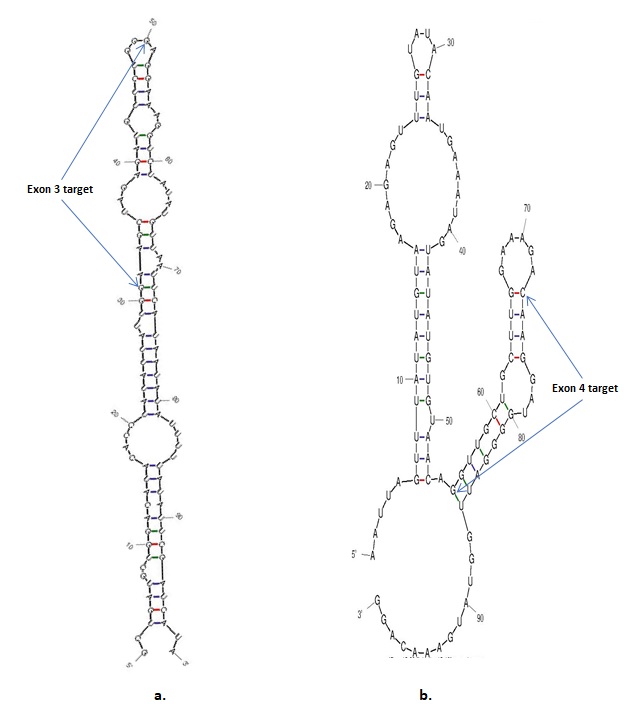


**Fig S7. The secondary structures of target *AcPDS* sgRNAs**

a. Secondary structure of AcPDS exon 3 target sgRNA

b. Secondary structure of AcPDS exon 4 target sgRNA

The secondary structures were analyzed using the program RNA Folding Form

(http://www.unafold.org/mfold/applications/rna-folding-form.php).

**Table S1: List of primers used in this study**

| **Primer Name** | **5’ to 3’ sequence** | **Amplicon size and region** | **PCR conditions** |
| --- | --- | --- | --- |
| *AcPDS* F | ATGAGTGTTATTGGATCGGTTTCTG | 4278 bp of full-length *AcPDS* gene | 1 cycle of 98 °C for 15 s; 35 cycles of 98 °C for 20 s, 58 °C for 20 s, 68 °C for 3 min, and 1 cycle of 68°C for 10 min |
| *AcPDS* R | CTAGACAGTCGTCATCTCAGCCT |  |  |
| *Cas9* F | TGAGCGACATCCTGAGAGTG | 610 bp of an internal sequence of *Cas9* | 1 cycle of 98 °C for 15 s; 35 cycles of 98 °C for 20 s, 58 °C for 20 s, 68 °C for 30 sec, and 1 cycle of 68°C for 10 min |
| *Cas9* R | TCGTTGGGCAGGTTCTTATC |  |  |
| *AcPDSg* F | GCTGATGCTGGACATACACC | 198 bp of *AcPDS* flanking both the targets | 1 cycle of 98 °C for 15 s; 35 cycles of 98 °C for 20 s, 58 °C for 20 s, 68 °C for 15 s, and 1 cycle of 68°C for 10 min |
| *AcPDSg* R | CCTGTTTCATACCAATCCCC |  |  |
